# The New York Medical Journal Changes Publishers

**Published:** 1900-09

**Authors:** 


### THE NEW YORK MEDICAL JOURNAL CHANGES PUBLISHERS.

THE *New York Medical Journal* so long published by D. Appleton & Company has passed into the hands of Mr. A. R. Elliott, the head of a prominent advertising house, who becomes sole owner and publisher. Those who have known the house of Appleton during all the years of its splendid career will keenly regret the circumstances that have led to this change.

It is hoped and believed that the new publisher will conduct the *Journal* on a high plane. The fact that Dr. Frank P. Foster is retained as editor indicates this purpose.

It has been an exponent of high class medical journalism for so long a period under its accomplished editor, that we take this occasion to offer to the new publisher congratulations upon his announced decision to retain Dr. Foster.

In writing of this change of publishers the New York *Medical Record* in a recent issue commented as follows:

> We understand . . . that the editorial department will remain in charge of the present editor, to whose able management for so many years the *Journal* owes its high professional standing.

In commenting further upon the change of publishers the *Medical Record* refers to the financial problems involved in the conduct of a medical journal as follows:

> It will doubtless be a matter for surprise to the great majority of the profession to learn that a medical journal apparently so prosperous was financially a failure, and it will perhaps dispel the idea which seems to prevail that the profits of publishing medical journals are fabulously large. Those who have had experience in such matters know well that the publication of a medical journal is very different from that of an illustrated magazine for the general public. The latter can be sold at a price irrespective of the cost of manufacture, and thereby secures an enormous circulation, the profit on the undertaking being derived wholly from the advertisements. The number and variety of the latter which can be secured for a monthly magazine are practically unlimited, whereas in the matter of reputable medical advertising there is a very sharply defined limit. This change of ownership of the *New York Medical Journal* may serve to impress the truth of the facts just stated upon the minds of more or less experienced financial backers of some other of our contemporaries which are now being published at a heavy loss.

Here is a hint that may well be pondered by subscribers. It ought to stimulate them to prompt payment of subscription bills.

Printers demand prompt payment, a fact that all considerate subscribers appreciate. The BUFFALO MEDICAL JOURNAL has much reason for satisfaction in this regard, for which it takes this occasion to thank its supporters most heartily.

